# The Role of Race and Insurance Status in Access to Genetic Counseling and Testing Among High-Risk Breast Cancer Patients

**DOI:** 10.1093/oncolo/oyac132

**Published:** 2022-09-16

**Authors:** Jihoon J Choi, Tsion Fikre, Alexandra Fischman, Anne K Buck, Naomi Y Ko

**Affiliations:** Boston Medical Center, Boston, MA, USA; Boston Medical Center, Boston, MA, USA; Graduate Medical Sciences, Boston University School of Medicine, Boston, MA, USA; Boston Medical Center, Boston, MA, USA; Graduate Medical Sciences, Boston University School of Medicine, Boston, MA, USA; Boston Medical Center, Boston, MA, USA; Department of Medicine, Section of Hematology Oncology, Boston University School of Medicine, Boston, MA, USA

## Abstract

**Purpose:**

The role of germline genetic testing in breast cancer patients is crucial, especially in the setting of the recent trials showing the benefit of PARP inhibitors. The goal of this study was to identify racial disparities in genetic counseling and testing in patients with high-risk breast cancer.

**Methods:**

Patients with 2 unique breast cancer diagnoses were examined to understand demographics, insurance coverage, characteristics of breast cancer, and whether they were recommended for and received genetic counseling and testing.

**Results:**

A total of 69 patients with a dual diagnosis of breast cancer between the years 2000 and 2017 were identified (42% identified as White compared to 58% that identified as non-White). White patients were more likely to be recommended for genetic counseling (OR = 2.85; 95% CI, 1.07-7.93, *P* < .05), be referred for genetic counseling (OR = 3.17; 95% CI, 1.19-8.86, *P* < .05), receive counseling (OR = 3.82; 95% CI, 1.42-10.83, *P* < .01), and undergo genetic testing (OR = 2.88; 95% CI, 0.97-9.09, *P* = .056) compared to non-White patients. Patients with private insurance were significantly more likely to be recommended for genetic counseling (OR 5.63, *P* < .005), referred (OR 6.11, *P* < .005), receive counseling (OR 4.21, *P* < .05), and undergo testing (OR 4.10, *P* < .05). When controlled for insurance, there was no significant racial differences in the rates of GC recommendation, referral, counseling, or testing.

**Conclusion:**

The findings of this study suggest that disparities in genetic counseling and testing are largely driven by differences in health insurance.

## Teaser

Genetic testing of breast cancer patients is necessary for optimal treatment. This article describes genetic testing patterns in a large urban safety-net hospital and evaluates the association between race and genetic testing in women with more than one unique breast cancer diagnosis.

**Implications for Practice**

The results of this study showed that a significant disparity exists in access to genetic testing for breast cancer patients, based on insurance coverage, even with these patients meeting the previously outlined criteria for genetic testing by the NCCN and ASCO. The analysis shows a significant difference in rates of recommendation, referral, and receipt of genetic counselling and testing when comparing White versus non-White patients.

## Introduction

The clinical landscape for genetic testing in breast cancer is changing. With the successful advent of targeted therapies such as PARP inhibitors in patients with BRCA mutations, the indication for genetic testing is rapidly expanding. Beyond the traditional role in identifying BRCA carriers for secondary prevention, genetic testing now has implications for treatment in both the adjuvant and metastatic settings.^,1-3^ These new findings suggest that genetic testing of breast cancer patients is necessary for optimal treatment.

Inherited genetic variations account for 5-10% of all female breast cancers and 15-20% of all familial breast cancers, with mutations most commonly seen in the *BRCA1* and *BRCA2* genes.^,4,5^ Patients with BRCA1 and BRCA2 mutations have a significantly higher risk of developing breast and ovarian cancer, and patients with a prior diagnosis of breast cancer have a higher risk of recurrence.^,6-9^

Given established risks in genetic mutation carriers and recent clinical trials that demonstrate the benefit of PARP inhibitors, it is ever more important to identify patients with BRCA mutations. Specifically, BRCA carriers who are aware of their status can lead to earlier screening, detection, and changes in treatment. The US Preventive Services Task Force (USPSTF) recommends that women with a personal or family history of breast cancer should be screened to determine if they need to be considered for genetic testing, and the National Comprehensive Cancer Network (NCCN) has established a set of guidelines for which patients would qualify for genetic testing.^10^

Despite the indications for genetic testing, studies suggest that patients who qualify for testing do not receive testing; only 29% of patients eligible for genetic testing noted that they discussed genetic testing with their providers, with only 15% ultimately undergoing testing.^11^ Genetic testing seems to be even less common among minority patients, even when barriers of ascertainment and cost were controlled^,12-15^

This study was designed to describe genetic testing patterns in a large urban safety-net hospital. The main objective of this study was to evaluate the association between race and genetic testing in women with more than one unique breast cancer diagnosis.

## Methods

This retrospective study included data from all patients who had 2 or more unique breast cancer diagnoses between 2000 and 2017. Cases were identified by the clinical data warehouse, and chart biopsies of every patient were conducted to ascertain that all patients included had 2 unique breast cancer diagnosis that were determined to not be a local recurrence. The study H-33680 was approved by the Institutional Review Board (IRB). Specific variables for each case were abstracted from the electronic medical record and a chart abstraction tool was created in Research Electronic Data Capture (REDCap).

Sociodemographic variables collected included age at each diagnosis, race, primary language, marital status, insurance coverage (private vs public), and family history of breast cancer. Staging information and characteristics of each breast cancer diagnosis were also collected for each breast cancer diagnosis, including histology, grade, receptor status, and the type of treatments that were received. Four outcomes were established: genetic counseling being recommended, genetic counseling referral being placed, receipt of counseling from a certified genetic counselor, and receipt of genetic testing. These primary outcomes were determined through chart biopsy of each patient and direct examination of clinical notes. The recommendation for genetic counseling was ascertained on whether a provider’s clinic note made mention of their discussion with patients regarding genetic counseling and whether it was recommended. In contrast, the referral for genetic counseling was noted if there was documentation that they would proceed with the genetic referral and the order was placed.

Demographic and clinical factors in both race groups, White and non-White, were analyzed using either Chi-square test for categorical variables or *t*-tests for continuous variables. Univariate and multivariate logistic regression was performed to assess the associations between race and the 4 outcomes, establish odds ratios with CIs and evaluate potential confounders. Statistical analysis was conducted using R software, version 1.2.1335.

## Results

**Figure 1.**
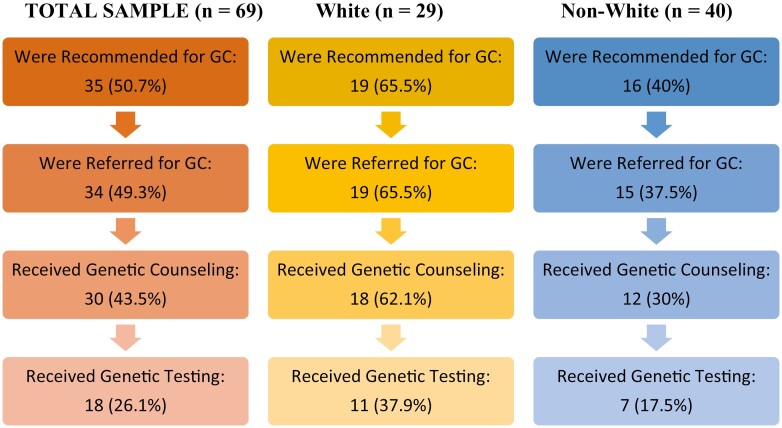
Flow diagram.

The study design and the four primary outcomes are shown in Figure 1. The demographics of the patients in this study are listed in Table 1. Of the 69 total patients included, 42% identified as White, compared to 58% that identified as non-White. The average age of White versus non-White patients was 72.7 years and 73.5 years, respectively, with no statistical difference in the age at the first and second diagnosis of breast cancer. The primary language in White patients was English in 89.7% of patients compared to 80% in non-White patients. There were no statistically significant differences in demographic and clinical variables across these 2 groups, except for the insurance type; in the White group, 48.3% of patients had private insurance with 51.7% public, compared to 12.5% private and 87.5% public in the non-White group (p <0.001).

**Table 1.** Socio-demographics by race

Characteristic	All patients (*n* = 69)	White (*n* = 29)	Non-White (n = 40)	*P*-value
Demographics				
Age, y—mean (SD)	73.17 (13.58)	72.72 (11.95)	73.5 (14.78)	.81
Age at first BRC diagnosis, years—mean (SD)	59.64 (12.82)	58.25 (10.85)	60.65 (14.12)	.43
Age at second BRC diagnosis, years—mean (SD)	62.4 (12.5)	61.8 (10)	62.8 (14.2)	.75
Primary language—no. (%)				
English	58 (84.1)	26 (89.7)	32 (80)	.45
Other	11 (15.9)	3 (10.3)	8 (20)	
Ethnicity—no. (%)				
Hispanic or Latino	6 (8.7)	0 (0)	6 (15)	.08
Not Hispanic or Latino	63 (91.3)	29 (100)	34 (85)	
Marital status—no (%)				
Partnered	26 (37.7)	11 (37.9)	15 (37.5)	>.999
Not partnered	43 (62.3)	18 (62.1)	25 (62.5)	
Insurance—no. (%)				
Private	19 (27.5)	14(48.3)	5(12.5)	.001
Public	50(72.5)	15(51.7)	35(87.5)	
Number of cancer diagnoses—no. (%)				
Third cancer diagnosis	17 (24.6)	7 (24.1)	10 (25)	>.999
Fourth cancer diagnosis	2 (7.7) [2/26]	0 (0)	2 (14.3) [2/14]	NA
Family history of cancer—no. (%)				
Breast cancer	15(55.6) [15/27]	6 (42.9) [6/14]	9 (69.2) [9/13]	.32
Ovarian cancer	3 (5.4) [3/56]	1 (4.8) [1/21]	2 (5.7) [2/35]	>.999

Outcomes	Total no. (%)	White no. (%)	Non-White no. (%)	*P* value
Did the provider recommend genetic counseling?	35 (50.7)	19 (65.5)	16 (40)	.04
Did the provider place a referral for genetic counseling?	34 (49.3)	19 (65.5)	15 (37.5)	.02
Did the patient receive counseling from a certified genetic counselor?	30 (43.5)	18 (62.1)	12 (30)	.008
Did the patient receive genetic testing?	18 (26.1)	11 (37.9)	7 (17.5)	.05

In Table 1, 65.5% of White patients were recommended for genetic counseling compared to 40% of non-White patients (*P* = 0.04). The percentage of White patients compared to non-White patients who were referred to a genetic counselor was 65.5% vs. 37.5% (*P* = .02). Those that received counseling from a licensed genetic counselor were 62.1% in White patients vs. 30% in non-White patients (*P* = .008). A total of 37.9% of White patients underwent genetic testing vs. 17.5% non-White patients (*P* = .05).

Univariate logistic regression models in Table 2 demonstrated that White patients were more likely to be recommended for genetic counseling (OR = 2.85; 95% CI, 1.07-7.93, *P* < .05), be referred for genetic counseling (OR = 3.17; 95% CI, 1.19-8.86, *P* < .05), receive counseling from a certified genetic counselor (OR = 3.82; 95% CI, 1.42-10.83, *P* < .01), and undergo genetic testing (OR = 2.88; 95% CI, 0.97-9.09, *P* = .05), in comparison to non-White patients. The age of patients at diagnosis was also associated with differences in the primary outcomes. When compared to those who received the second diagnosis after the age of 70, patients below the age of 70 had significantly increased odds of being recommended for genetic counseling (OR 9.48, *P* = .0004), getting referred to GC (OR 8.70, *P* = .006), and receiving genetic counseling (OR 6.26, *P* = .004), although without a significant difference in getting tested (OR 4.0, 95% CI, 0.98-27.16, *P* = .07).

**Table 2.** Univariate logistic regression

	OR (95% CI)	*P* value
Genetic counseling recommendation		
Race		
Non-White	Reference	
White	2.85 (1.07, 7.93)	.04
Age category		
70 +	Reference	
Under 70	9.48 (2.72, 44.86)	.0004
Insurance		
Public	Reference	
Private	5.63 (1.75, 22.02)	.004
Genetic counseling referral		
Race		
Non-White	Reference	
White	3.17 (1.19, 8.86)	.02
Age category		
70 +	Reference	
Under 70	8.70 (2.50, 41.08)	.006
Insurance		
Public	Reference	
Private	6.11 (1.90, 24.01)	.002
Counseling from a certified genetic counselor		
Race		
Non-White	Reference	
White	3.82 (1.42, 10.83)	.008
Age category		
70 +	Reference	
Under 70	6.26 (1.81, 23.39)	.004
Insurance		
Public	Reference	
Private	4.21 (1.41, 13.86)	.01
Receive genetic testing		
Race		
Non-White	Reference	
White	2.88 (0.97, 9.09)	.05
Age category		
70+	Reference	
Under 70	4.00 (0.98, 27.16)	.07
Insurance		
Public	Reference	
Private	4.10 (1.30, 13.37)	.01

Univariate logistic modeling also demonstrated a significant difference when examining insurance types for patients. When compared to patients with public insurance, patients with private insurance were significantly more likely to be recommended for genetic counseling (OR 5.63, *P* = .004), referred (OR 6.11, *P* = .002), receive counseling (OR 4.21, *P* = .01), and undergo testing (OR 4.10, *P* = .01).

However, multivariate logistic regression models in Table 3 demonstrated that, when controlling for insurance and age, the race was not a statistically significant predictor for any of the primary outcomes. There was no statistical difference between non-White and White patients in being recommended for GC (OR 1.49, *P* = .51), being referred to GC (OR 1.69, *P* = .38, receiving counseling (OR 2.45, *P* = .12), and undergoing testing (OR 1.72, *P* = .39).

**Table 3.** Multivariate logistic regression

	OR (95% CI)	*P* value
Genetic counseling recommendation		
Adjusted by insurance		
Non-White	Reference	
White	1.84 (0.61, 5.50)	.27
Private insurance	4.43 (1.27, 18.32)	.02
Adjusted by age and insurance		
Non-White	Reference	
White	1.49 (0.45, 4.93)	.51
Private insurance	4.61 (1.18, 22.30)	.04
Age <70 years	8.78 (2.32, 45.25)	.003
Genetic counseling referral		
Adjusted by insurance		
Non-White	Reference	
White	2.03 (0.67, 6.11)	.21
Private insurance	4.67 (1.34, 19.28)	.02
Adjusted by age and insurance		
Non-White	Reference	
White	1.69 (0.51, 5.55)	.38
Private insurance	4.83 (1.25, 23.00)	.03
Age <70 years	7.99 (2.09, 41.60)	.005
Counseling from a certified genetic counselor		
Adjusted by insurance		
Race		
Non-White	Reference	
White	2.76 (0.94, 8.32)	.07
Private insurance	2.87 (0.86, 10.08)	.09
Adjusted by age and insurance		
Race		
Non-White	Reference	
White	2.45 (0.79, 7.76)	.12
Private insurance	2.69 (0.77, 10.07)	.13
Age <70 years	5.23 (1.41, 25.84)	.02
Received genetic testing		
Adjusted by insurance		
Race		
Non-White	Reference	
White	1.92 (0.56, 6.62)	.29
Private insurance	3.16 (0.91, 11.26)	.07
Adjusted by age and insurance		
Race		
Non-White	Reference	
White	1.72 (0.49, 6.02)	.39
Private insurance	2.97 (0.84, 10.77)	.09
Age <70 years	3.16 (0.72, 22.21)	.17

Staging information for cancer diagnosis was also examined to identify whether there were significant differences in rates of genetic recommendation, referral, counseling, and testing across cancer stages at second diagnosis, as seen in Table 4. Of the 69 total patients included in the study, staging information was complete or available in 59 patients. There was no significant difference in rates of recommendation (*P* = .894), referral (*P* = .780), receipt of genetic counseling (*P* = .654), or genetic testing (*P* = .571) across the stage of second diagnosis of breast cancer. There was also no difference in staging across insurance coverage (*P* = .539) and race (*P* = .818).

**Table 4.** Cancer staging

Pathologic stage—*N* = 59	Provider recommend genetic counseling—yes	Provider recommend genetic counseling—no	*P* value
Stage 0	6	7	.894
Stage I	13	12	
Stage II	8	5	
Stage III	4	2	
Stage IV	0	2	

Pathologic stage—*N* = 59	Referral for genetic counseling—yes	Referral for genetic counseling—No	*P* value
Stage 0	6	7	.780
Stage I	13	12	
Stage II	8	5	
Stage III	3	3	
Stage IV	0	2	

Pathologic stage—*N* = 59	Patient received genetic counseling—Yes	Patient Received genetic counseling—No	*P* value
Stage 0	4	9	.654
Stage I	12	13	
Stage II	8	5	
Stage III	3	3	
Stage IV	0	2	

Pathologic stage—*N* = 59	Patient received genetic testing—Yes	Patient received genetic resting—No	*P* value
Stage 0	4	9	.571
Stage I	6	19	
Stage II	5	8	
Stage III	1	5	
Stage IV	0	2	

Pathologic stage—*N* = 59	Private insurance	Public insurance	*P* value
Stage 0	5	8	.539
Stage I	6	19	
Stage II	6	7	
Stage III	2	4	
Stage IV	0	2	

Pathologic stage—*N* = 59	White	Non-White	*P* value
Stage 0	4	9	.818
Stage I	11	14	
Stage II	7	6	
Stage III	3	3	
Stage IV	1	1	

## Discussion

As germline genetic testing has become essential in the treatment of breast cancer, we sought to describe the patterns in genetic counselling and testing among a racially diverse patient population. Over 17 years, among a diverse patient population seeking care at a large urban safety net hospital, we found that patients from non-White racial backgrounds were less likely to be recommended for genetic counseling, be referred to a genetic counselor, receive counseling from a genetic counselor, and ultimately undergo genetic testing when compared to White patients. However, when controlling for insurance coverage, these differences were no longer statistically significant, which is suggestive that insurance coverage has been a driving factor for disparities in genetic counseling and testing.

To our knowledge, this study is the first retrospective analysis to demonstrate that insurance is the primary driver for the disparities that exist in genetic counseling and testing among racially diverse patients with breast cancer. Previous literature in this field had noted significant disparities in genetic testing disparities due to race.^,12,13,16^ Cragun et al did note the impact of having private insurance on both genetic testing discussion and receipt of testing; however, their analysis showed that race still played a role even after controlling for socioeconomic factors such as insurance.^14^

Disparities in healthcare outcomes due to differences in insurance coverage in cancer patients have been well documented in the literature. Ellis et al examined 5-year all-cause and cancer-specific survival rates from the California Cancer Registry and found that the improvements in cancer survival rates from 1997 to 2014 in patients with breast, prostate, colorectal, lung cancer, or melanoma were almost exclusively limited to patients with private health insurance.^17^ Recent literature also reveals differences in healthcare outcomes in breast cancer patients specifically; Hsu et al found that compared to patients with private insurance, those without private insurance were significantly more likely to have both a late-stage breast cancer diagnosis and increased risk of death from breast cancer compared to those with Medicaid or no insurance coverage. Ko et al found that White patients were less likely to receive a diagnosis of locally advanced breast cancer compared to Blacks, Hispanics, and other minority groups, and almost half of these differences in stage at presentation across racial groups could be attributed to differences in healthcare coverage.^18,19^ Similarly, in a study that examined the California Cancer registry, Miguel et al found that compared to breast cancer patients with private health insurance, patients in other insurance groups had higher mortality regardless of age group.^20^ These differences in mortality rates and health care are in part likely due to differences in preventative screening, although prior studies have also shown that breast patients with Medicaid receive different and less aggressive treatments.^21,22^

Prior studies have also shown varying rates of genetic testing discussions, referrals, and testing between providers and patients. Childers et al found the overall rates of genetic counseling recommendation and testing for patients with newly diagnosed breast cancer of 20.2% and 15.3%, respectively, compared to a study by Kurian et al found rates of 71% and 53% in high-risk patients, respectively.^11,23^ Overall, our results are comparable to the existing literature, and variances in these rates are likely due to multiple factors including differences in study design, patient sampling, and the time period during which patients were studied. Studies have previously shown increasing rates in testing in recent years, likely due to the guidelines for testing by the USPTF and NCCN, as well as the initiation of the Affordable Care Act (ACA) which mandated coverage for preventative services recommended by the USPSTF.^24,25^

Healthcare outcomes and survival benefits do not arise from testing itself, but rather from changes in management and screening following a positive genetic test. Expanding availability and access to care have previously been shown in the literature to have significant, measurable outcomes in breast cancer screening. For example, Toyoda et al found that expanded Medicaid eligibility and coverage under the ACA noted an increase in screening mammogram rates in women from low-income households compared to states that did not expand coverage, while Le Blanc et al found that an association in Medicaid expansion and reduced incidence of advanced-stage breast cancer at diagnosis, especially in African American patients.^26,27^ We suspect from our findings that expanding coverage for genetic testing and counseling would increase screening rates and detection of breast cancers, which would have significant clinical outcomes for all patients.

Expanding coverage for genetic testing would also help clarify the role of contralateral prophylactic mastectomy (CPM) in breast cancer patients. While there has been shown to be no significant benefit for CPM in average-risk patients and is not recommended by the American Society of Breast Surgeons (ASBrS), their guidelines note that CPM should be considered for those at high risk for contralateral breast cancer, which includes documented BRCA mutation carriers, as studies have shown that CPM did increase overall survival for patients with BRCA mutations.^28-31^. Metcalfe et al recently found that patients that received a negative result were significantly less likely to choose a prophylactic bilateral mastectomy.^32^ However, despite the role that genetic testing plays in surgical treatment decisions, Armstrong et al recently found that of patients who had genetic testing within a year of diagnosis, almost half (45.6%) of patients did not receive their results before surgery and that a negative result was associated with significantly lower rates for bilateral mastectomies over the age of 50.^33^

Our study did try to explore the rates for the various steps a patient must go through before undergoing genetic testing. By breaking down this process into these levels, we hoped to understand the different barriers that may exist at each level, which in turn would open avenues for possible targeted interventions. This type of analysis was limited by our smaller sample size, although future research will be directed toward identifying these barriers to help develop multi-level interventions to address the disparities present in genetic testing.

The results from this study should be considered within its limitations. Our sample size is small given that our scope was based on patients with 2 separate diagnoses of breast cancer—this is just one subgroup of patients that had qualified for genetic testing during this time. Our study was conducted with data from an urban safety-net hospital within an academic center and may not be generalizable to other healthcare settings. The demographics of this patient population were by self-report, and we relied on the EMR to define race. The analysis was based on the retrospective review of clinical documentation, which does not encompass the shared decision-making that physicians may have had with their patients, such as in the recommendation or referral for genetic testing. Within our analysis, we were also unable to account for any patients that were lost to follow up after the second breast cancer diagnosis. In addition, rates for genetic counseling and testing may be lower if patients chose to seek care outside of the electronic healthcare record.

Overall, our study demonstrates that insurance played a large role among a diverse patient population in determining whether genetic counseling and testing were considered and completed. Healthcare coverage to include genetic testing in breast cancer is critical for optimal treatment based on new and developing breast cancer treatment options to improve disease recurrence and survival. Further studies to identify racial disparities in genetic testing will require prospective studies to determine whether barriers continue to exist in genetic counseling independent of insurance.

## Data Availability

The data underlying this article will be shared on reasonable request to the corresponding author.
